# Elevated *NRAS* expression during DCIS is a potential driver for progression to basal-like properties and local invasiveness

**DOI:** 10.1186/s13058-022-01565-5

**Published:** 2022-10-18

**Authors:** Ze-Yi Zheng, Hanan Elsarraj, Jonathan T. Lei, Yan Hong, Meenakshi Anurag, Long Feng, Hilda Kennedy, Yichao Shen, Flora Lo, Zifan Zhao, Bing Zhang, Xiang H.-F. Zhang, Ossama W. Tawfik, Fariba Behbod, Eric C. Chang

**Affiliations:** 1 Lester and Sue Smith Breast Center and Dan L. Duncan Comprehensive Cancer Center, Baylor College of Medicine, Houston, TX 77030 USA; 2 Department of Medicine, Baylor College of Medicine, Houston, TX 77030 USA; 3 Department of Pathology and Laboratory Medicine, University of Kansas Medical Center, Kansas City, KS 66160 USA; 4 Department of Pathogenic Organism Biology, Henan University of Chinese Medicine, Zhengzhou, People’s Republic of China; 5 Cancer Cell Biology Graduate Program, Baylor College of Medicine, Houston, TX 77030 USA; 6 Department of Molecular and Cellular Biology, Baylor College of Medicine, Houston, TX 77030 USA; 7 MAWD Pathology Group, St. Luke’s Hospital, Lenexa, KS 66215 USA

**Keywords:** Breast cancer, DCIS, Premalignancy, Invasion, Ras GTPase

## Abstract

**Background:**

Ductal carcinoma in situ (DCIS) is the most common type of in situ premalignant breast cancers. What drives DCIS to invasive breast cancer is unclear. Basal-like invasive breast cancers are aggressive. We have previously shown that NRAS is highly expressed selectively in basal-like subtypes of invasive breast cancers and can promote their growth and progression. In this study, we investigated whether *NRAS* expression at the DCIS stage can control transition from luminal DCIS to basal-like invasive breast cancers.

**Methods:**

Wilcoxon rank-sum test was performed to assess expression of *NRAS* in DCIS compared to invasive breast tumors in patients. *NRAS* mRNA levels were also determined by fluorescence in situ hybridization in patient tumor microarrays (TMAs) with concurrent normal, DCIS, and invasive breast cancer, and association of *NRAS* mRNA levels with DCIS and invasive breast cancer was assessed by paired Wilcoxon signed-rank test. Pearson’s correlation was calculated between *NRAS* mRNA levels and basal biomarkers in the TMAs, as well as in patient datasets. RNA-seq data were generated in cell lines, and unsupervised hierarchical clustering was performed after combining with RNA-seq data from a previously published patient cohort.

**Results:**

Invasive breast cancers showed higher *NRAS* mRNA levels compared to DCIS samples. These *NRAS*^*high*^ lesions were also enriched with basal-like features, such as basal gene expression signatures, lower ER, and higher p53 protein and Ki67 levels. We have shown previously that NRAS drives aggressive features in DCIS-like and basal-like SUM102PT cells. Here, we found that *NRAS*-silencing induced a shift to a luminal gene expression pattern. Conversely, *NRAS* overexpression in the luminal DCIS SUM225 cells induced a basal-like gene expression pattern, as well as an epithelial-to-mesenchymal transition signature. Furthermore, these cells formed disorganized mammospheres containing cell masses with an apparent reduction in adhesion.

**Conclusions:**

These data suggest that elevated NRAS levels in DCIS are not only a marker but can also control the emergence of basal-like features leading to more aggressive tumor activity, thus supporting the therapeutic hypothesis that targeting NRAS and/or downstream pathways may block disease progression for a subset of DCIS patients with high *NRAS*.

**Supplementary Information:**

The online version contains supplementary material available at 10.1186/s13058-022-01565-5.

## Background

Ductal carcinoma in situ (DCIS) is the most common form of noninvasive breast cancers. In DCIS, cancer cells have expanded inside the breast ducts; however, they have not yet broken through the basement membrane and the myoepithelium to become invasive. The current standard of care for DCIS includes surgery by mastectomy or lumpectomy plus radiation, and endocrine therapy (for hormone receptor-positive DCIS). Although additional radiotherapy and endocrine therapy have resulted in significant improvement in prevention of invasive breast cancer recurrence, they have not resulted in improved patient survival. Thus, the 20-year breast cancer mortality rate following a DCIS diagnosis, with or without additional radiation and endocrine therapy, remains at 3.3% [1].

Studies that have investigated the natural progression of human DCIS have reported untreated DCIS to progress to invasive breast cancer at a rate of approximately 40% [2]. Untreated DCIS are those that were originally misdiagnosed with benign breast diseases but had subsequent examination with DCIS or those with biopsy proven DCIS who underwent non-operative management [3–5]. The risk factors significantly associated with the development of invasive disease are high grade, calcifications, younger age (< 60 years), absence of endocrine therapy, and lesion size [2, 6]. Currently, two diagnostic tests have been developed for risk stratification in DCIS, DCIS Score™, and DCISionRT [7–9]. These tools have not yet been widely adopted, and their clinical utility remains unclear.

Basal-like breast cancers account for 10–20% of all invasive breast cancers, and they are highly proliferative and very aggressive [10]. These tumors are called “basal-like” because they express markers of the basal region in the mammary structure. These basal-like tumors are usually, but not always, triple-negative for ER, PR, and HER2. Interestingly, premalignant lesions prior to DCIS (such as atypical ductal hyperplasia, ADH) are nearly all ER^+^, and basal-like lesions first appear at DCIS at a frequency of 4–8% [11]. Furthermore, it has been noted for some time that patients whose tumors are initially ER^+^ can later become ER^–^ when they relapse after endocrine therapy [12]. These data suggest that luminal breast tumor cells have a great degree of plasticity, and that they can evolve into cancer cells with basal-like properties during DCIS progression, which may lead to the emergence of invasive breast cancers.

The Ras superfamily of GTPases are encoded by three *RAS* genes, *HRAS*, *NRAS*, and *KRAS*. Oncogenic *RAS* mutations, which lock the RAS proteins in the GTP-bound state, can be found in approximately 30% of human cancers [13]; however, *RAS* oncogenic mutations are very rare in primary breast cancer [14]. Instead, we have shown previously that *wild-type* NRAS is selectively overexpressed in the basal-like breast cancers [15]. By gene silencing, *NRAS* has been demonstrated to be necessary for the growth of basal-like breast cancer cells but not that of luminal breast cancer cells or the closely related claudin-low cells [15]. More importantly, we have evidence that NRAS overexpression can promote more aggressive tumor activity, such as tumor formation in mice when co-transplanted with fibroblasts [15]. In support of these findings, a more recent clinical study of 198 previously untreated breast cancer patients with long-term follow-up showed that among the RAS family members, *NRAS* expression was significantly associated with triple negativity, higher grade, and reduced overall and disease-free survival. In the multivariable analysis, elevated *NRAS* mRNA independently predicted reduced overall and disease-free survival [16].

In this study, we investigated whether NRAS is responsible for the emergence of basal-like properties during DCIS and invasiveness. Our results suggest that N-Ras is not only a biomarker for invasiveness that it can also drive the emergence of basal-like properties from luminal cells.

## Methods

### Cell culture media and general reagents

SUM102PT and SUM102PT cells carrying DOX-inducible shRNA against *NRAS* were as described previously [15]. SUM225 cells were cultured in DMEM/F12 medium (Thermo Fisher Scientific) supplemented with 5% fetal bovine serum (Sigma-Aldrich), 10 mM HEPES (Thermo Fisher Scientific), non-essential amino acids (Thermo Fisher Scientific), 5 µg/mL human insulin (Sigma-Aldrich), and 1 µg/mL hydrocortisone (Sigma-Aldrich).

### Mammosphere formation assay

DOX-inducible *N-RAS* overexpressed SUM225 cells used in mammosphere experiment were created by transiently infect the cells with lentivirus carrying pINDUCER-NRAS as previously described [15]. Single cells were plated on ultra-low attachment 24-well plates (Corning) at a density of 5,000 viable cells per well. Cells were grown in serum-free mammary epithelial basal medium (Lonza) supplemented with 20 ng/ml EGF, 5 µg/ml insulin, 1 µg/ml hydrocortisone (Lonza), 20 ng/ml β-FGF, B27 (Invitrogen), 4 µg/ml heparin (MP Biomedicals), and Antibiotic–Antimycotic (Invitrogen). Cells were fed every three days by adding an additional media (10% of total volume) to the wells. When noted, 2 µg/ml DOX was also added. Mammospheres were cultured for 1.5 or 3 months before being examined and photographed on an Olympus IX71 microscope. The numbers of abnormal vs normal spheres were counted in a double-blind fashion.

### Detection of *NRAS* expression in TMAs by FISH

TMAs were constructed from paraffin-embedded, formalin-fixed (FFPE) sections of breast tissue from patients diagnosed with concurrent DCIS and IDC (*n* = 22). The samples in the TMAs were obtained from individuals enrolled under an IRB-approved protocol and following US Common Rule.

FISH procedures were performed using RNAscope^®^ Probe-Hs-NRAS (Advanced Cell Diagnostics), RNAscope^®^ Negative Control Probe-DapB (Advanced Cell Diagnostics), and RNAscope Multiplex Fluorescent Reagent Kit V2 (Advanced Cell Diagnostics) on FFPE sections, according to manufacturer’s instructions. Data analysis was performed using ImageJ. Each fluorophore channel was analyzed separately. The region of interest (ROI) was selected around the DCIS or invasive lesions. A number of cells in ROI were quantified by counting Hoechst positive nuclei (number of nuclei/ROI). FISH signals were then analyzed in the same ROI (number of signals/ROI). The “average number of signal/cell” was calculated by dividing “number of signals/ROI” by “number of nuclei/ROI.” At least three images per patient were analyzed.

### qPCR

RNA was isolated using RNeasy kit (Qiagen), and cDNAs were synthesized using SuperScript IV First-Strand Synthesis System (Thermo Fisher Scientific). Real-time PCR was conducted with Power SYBR Green Master Mix (Thermo Fisher Scientific) on a CFX Real-Time PCR system (Bio-Rad). The primers (5′- > 3′) used were: *NRAS*, forward: TGGTGGTTGGAGCAGGTG; reverse: GCCTTCGCCTGTCCTCATGTA. *KRT8*, forward: AGCGTACAGAGATGGAGAACGA; reverse: AGCTCCCGGATCTCCTCTTC. *ACTB*, forward: CACCATTGGCAATGAGCGGTTC; reverse: AGGTCTTTGCGGATGTCCACGT. The relative amounts of PCR products generated from each primer set were determined on the basis of threshold cycle (Ct) using *ACTB* as the loading control.

### RNA-seq

SUM102PT carrying DOX-inducible shRNA against *NRAS* [15] was seeded with or without 2 µg/ml DOX for 3 months. To maintain *NRAS* silencing, fresh DOX was added when the medium was replenished. To overexpress NRAS, pBABE-NRAS [15] or pBABE vector control was used to transduce SUM225 cells, which were then selected in 1 µg/ml puromycin for 2 days. The resulting cells were grown in puromycin-free medium for five weeks. One µg of total RNA isolated by RNeasy Mini Kit (QIAGEN) was sent to Novogene for RNA quality control (Agilent 2100 Bioanalyzer), library preparation, and next-generation sequencing (Illumina NovaSeq 6000). For RNA-seq processing, paired-end 150 bp reads were aligned to hg19 (GRCh37) reference genome using RSEM v1.2.31 [17] and Bowtie 2 [18]. Log2 RSEM counts of protein-coding genes were upper quantile normalized and used for downstream analysis. The RNA-seq data have been submitted to the GEO public database (GSE215407).

### Breast cancer patient transcriptomic profiling datasets

Two published breast cancer microarray datasets (GSE59248 and GSE26304) were used for *NRAS* expression analysis. They were downloaded from NCBI Gene Expression Omnibus (https://www.ncbi.nlm.nih.gov/geo/) as matrix files in txt format. In GSE59248, microarray was performed on Agilent-028004 SurePrint G3 Human GE 8 × 60 K Microarray Platform (GPL13607) and mean values of all probes mapping to a gene were taken for downstream analysis. In GSE26304, microarray was performed on Agilent-012391 Whole Human Genome Oligo Microarray G4112A (GPL6848), and there was one probe for *NRAS* without replicates. In addition, RNA-seq from a CPTAC breast cancer study was used [19].

### Statistical analysis

Pearson correlation coefficients in Table 1 were calculated between *NRAS* mRNA expression levels for each breast biomarker (ER, PR, HER2, Ki67, and P53) by correlating the average *NRAS* mRNA signal per cell and biomarker expression in each DCIS and IDC patient.

**Table 1:** Up-regulation of *NRAS* expression levels correlates with features found commonly in basal-like tumors

	*NRAS* versus ER^1^, *n* = 21		*NRAS* versus PR, *n* = 21		*NRAS* versus HER2, *n* = 16		*NRAS* versus Ki67, *n* = 20		*NRAS* versus p53, *n* = 17	
	DCIS	IDC	DCIS	IDC	DCIS	IDC	DCIS	IDC	DCIS	IDC
Pearson Correlation:	–0.49	–0.52	–0.31	0.09	0.24	0.3	0.55	0.68	0.54	0.55
*P* (two-tailed):	0.0341	0.0148	0.2133	0.7012	0.3991	0.2563	0.0187	0.0009	0.0364	0.0233

^1^ER, HER2, Ki67, and p53 levels were measured by IHC. *NRAS* levels were assessed by FISH. See Additional file 1: Table S1 for the levels of biomarkers

All RNA-seq statistical analysis was performed in R (version 4.0.2). The R package limma [20] was used to compute differences by moderated t-test for each gene. Signed –log10 p values from limma analyses were used as input for Gene Set Enrichment Analysis using default parameters with WebGestalt [21]. Specific methods used to calculate p values are also described in each figure legend. To combine RNA-seq data from breast tumors in the CPTAC cohort [19], RSEM-normalized log2-transformed counts were combined with cell line data generated in this study. The entire dataset was then batch corrected with ComBat-seq [21] before clustering using the top 1,000 genes with highest variance across the combined dataset with ComplexHeatmap [22] with the following parameters: clustering_distance_columns = “spearman,” clustering_distance_rows = “euclidean,” clustering_method_rows = “ward.D2.”

## Results

### High *NRAS* expression levels in DCIS samples from patients correlate with invasion

**Fig. 1:**
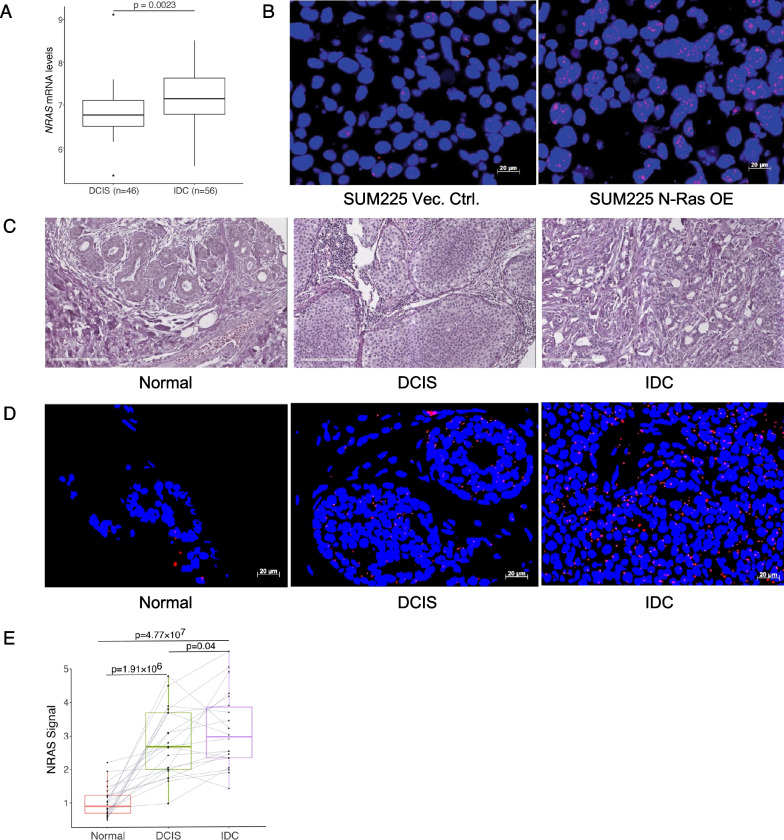
Up-regulation of *NRAS* expression levels correlates with progression to invasive breast cancer from DCIS. **A** Microarray data (GSE59248) from 46 DCIS and 56 invasive ductal carcinoma (IDC) samples were analyzed by the two-sided Wilcoxon rank-sum test. **B** SUM225 cells carrying either a vector control or an expression vector for *NRAS* (red dots) were fixed and probed by an *NRAS*-specific sequence and visualized using fluorescence microscopy. **C** The TMAs examined in this study have concurrent DCIS and IDC as assessed by H&E staining. **D** Representative images of *NRAS* mRNA FISH (red) on a patient tissue microarray with concurrent DCIS and IDC lesions. Nuclei were counterstained by DAPI (blue). **E** The RNA signals from panel-D and 21 additional samples like this were quantified and analyzed by Wilcoxon signed-rank test (paired)

To assess whether N-Ras can control progression during DCIS, we first examined a microarray data set derived from a study comparing gene expression levels in DCIS vs. invasive breast cancer [23]. Our analysis revealed that *NRAS* mRNA levels were significantly higher in invasive breast tumors than in DCIS (Fig. 1A).

Next, we took an orthogonal approach to study the relationship between *NRAS* mRNA levels and DCIS progression. NRAS-specific antibodies are not available for robust analyses of clinical samples [24], and we thus performed fluorescence in situ hybridization (FISH) on tissue microarrays (TMAs) consisting of 22 concurrent DCIS/IDC lesions, as well as adjacent normal tissues (Additional file 1: Table S1). Using an *NRAS*-specific probe (Fig. 1B), the FISH data were quantified to show that *NRAS* mRNA levels are significantly higher in DCIS than in the normal regions (Fig. 1C, E). Moreover, *NRAS* levels are higher in IDC than in DCIS regions (Fig. 1C, E), suggesting a progressive increase of expression from normal, DCIS, to IDC.

### *NRAS* expression correlated with basal-like features in DCIS patient samples

**Fig. 2:**
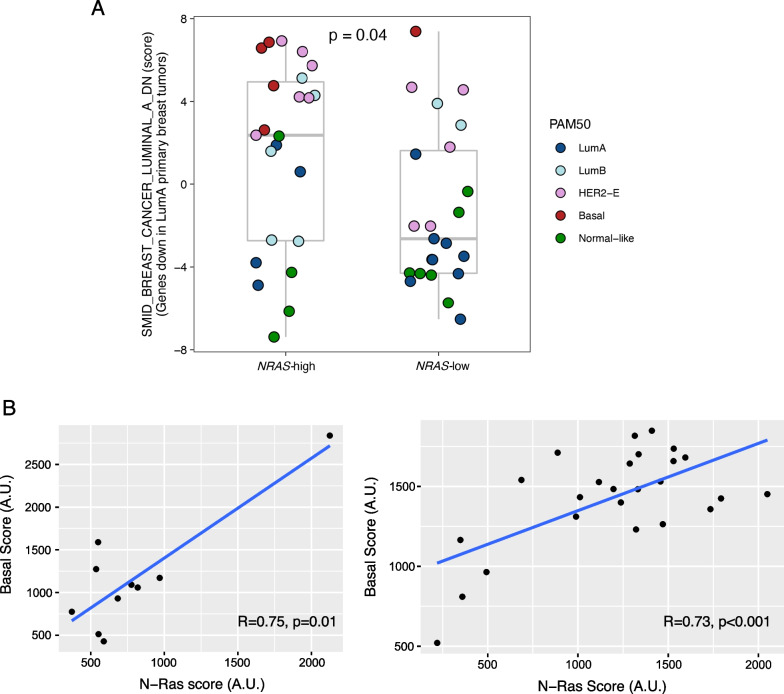
Expression of *NRAS* correlates with basal-like features in DCIS patient samples. **A** DCIS tumors in microarray data set GSE59248 as described in Fig. 1A were stratified by *NRAS* mRNA levels according to median expression. Single sample GSEA (ssGSEA) scores for the SMID_BREAST_CANCER_LUMINAL_A_DN signature from MSigDB computed using ssGSEA2.0 [41]. P values were derived from Wilcoxon rank-sum tests comparing ssGSEA scores in *NRAS-*high vs low samples. Intrinsic molecular subtypes in these tumors were determined by PAM50 as annotated in the GSE59248 dataset. **B** Pearson correlation analysis was performed to assess the relationship between an NRAS gene expression score and a basal gene expression score in DCIS patients. Shown on the left is a microarray dataset GSE59248 (*n* = 10), while RNA-seq dataset GSE33692 is shown on the right (*n* = 25)

To investigate the role of NRAS in the emergence of basal-like properties during DCIS, we first analyzed the same microarray dataset as described in Fig. 1A to determine whether *NRAS* mRNA levels were associated with basal-like properties. We examined a dataset [25] for a gene signature that is down-regulated in primary luminal-A tumors, as compared to basal tumors. The data show that this luminal-A down-regulated gene signature is expressed at significantly higher levels in DCIS samples with high *NRAS* levels (Fig. 2A). We note that the great majority of DCIS tumors displayed basal properties, as determined by PAM50 [26], also have higher levels of *NRAS* mRNA (see below for more analysis).

We have previously uncovered a gene signature associated with *NRAS* expression in basal-like breast cancer [15]. As shown in Fig. 2B, we applied this signature as an orthogonal approach to assess the role of NRAS signaling in two additional DCIS cohorts [27, 28] and found a significant correlation with this basal-like gene signature [29].

Finally, we further investigated the relationship between *NRAS* expression levels and basal-like properties in the aforementioned TMA sample set. These patient concurrent DCIS/IDC samples were evaluated for the expression of clinically relevant biomarkers including ER, PR, HER2, Ki67 and P53 (Additional file 1: Table S1). A correlation between the expression of these biomarkers and *NRAS* expression was performed using Pearson correlation. These studies showed that among the evaluated biomarkers, *NRAS* expression showed a significant correlation with basal-like features: low ER and high Ki67 (Table 1). Furthermore, basal-like tumors frequently carry *TP53* mutations [30]. Wild-type p53 has a very short half-life; thus, it is usually undetectable by IHC; however, mutant p53 protein levels can be more readily detected by IHC [31]. We observed higher p53 levels in *NRAS*^*high*^ tumors (Table 1), agreeing with the concept that these tumors display basal properties. These included both the DCIS and IDC regions of concurrent DCIS/IDC samples. Collectively, these clinical data support the concept that NRAS plays a key role in the emergence of basal-like high proliferative features during DCIS-IDC transition.

### *NRAS*-silencing in basal DCIS-like cells induces a luminal gene expression pattern

**Fig. 3:**
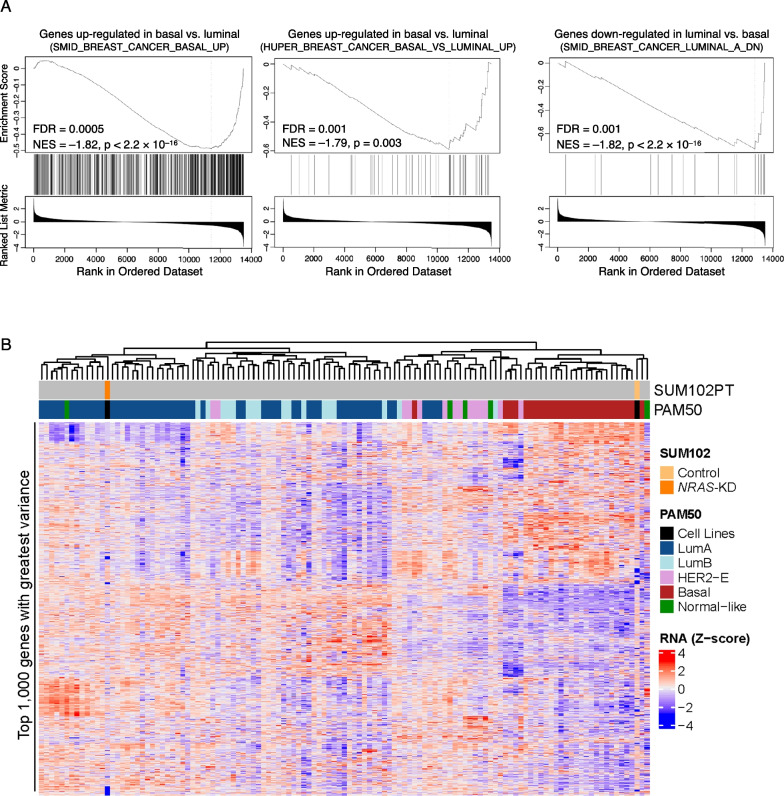
*NRAS*-silencing induced a switch from basal to luminal gene expression pattern in SUM102PT cells. **A** SUM102PT cells carrying a DOX-inducible shRNA against *NRAS* were seeded with or without DOX and cultured for 3 months. mRNAs from these cells were analyzed by RNA-seq, and GSEA was performed for previously published signatures containing genes known to be differentially expressed in luminal versus basal breast tumors. **B** The RNA-seq data generated from SUM102PT cells and from a CPTAC breast cancer cohort were combined, and then, the top 1000 most variable genes across combined samples were used to perform unsupervised hierarchal clustering

The clinical data demonstrated a strong correlation between up-regulation of *NRAS* in the mostly luminal DCIS and the emergence of basal-like properties and more aggressive tumor activities, such as higher level of proliferation and invasion. To ascertain whether *NRAS* plays a more direct role in controlling the balance between luminal to basal properties during DCIS, we turned to a basal-like and DCIS-like cell line model, SUM102PT [32]. We have shown previously that this cell line has high levels of NRAS (as compared to cell lines of other breast cancer subtypes) and NRAS can promote tumor growth of these cells in vivo [15]. In this study, we knocked down (KD) *NRAS* expression using a DOX-inducible shRNA clone as reported previously [15], and mRNAs were harvested over a four-month period. DOX was replenished when medium was changed to maintained *NRAS* silencing (Additional file 2: Fig. S1). To select the appropriate time points for RNA-seq, we performed qPCR to measure the expression of a luminal marker, *CK8/KRT8* (Additional file 2: Fig. S1). We thus generated RNA-seq data on these SUM102PT cells after three months in the presence of DOX (Additional file 3: Table S2) and performed Gene Set Enrichment Analysis (GSEA) to analyze datasets derived from examining differential gene expression between luminal and basal breast cancer cells (Additional file 3: Table S2). We found that genes that are down-regulated in *NRAS*^*KD*^ SUM102PT cells matched genes that are known to be up-regulated in basal breast cancer cells in at least two datasets [25, 33] (Fig. 3A). Consistent with the possibility that these genes are enriched in basal-like cells, they were down-regulated in luminal breast cancer cells as seen in a previous study [25] (Fig. 3A). These results support the hypothesis that *NRAS*-silencing reduced expression of genes typically up-regulated in basal-like breast cancer cells.

Next, the SUM102PT RNA-seq data were combined with RNA-seq data from a recent Clinical Proteomic Tumor Analysis Consortium (CPTAC) breast cancer cohort study [19]. Top 1,000 most variable genes (Additional file 3: Table S2) were used to perform unsupervised hierarchal clustering. Our data showed that while the *NRAS*^+^ SUM102PT control cells clustered together with tumors of the basal-like subtype as expected, the *NRAS*-silenced set from these cells clustered with the luminal-A subtype (Fig. 3B).

### *NRAS* overexpression in luminal DCIS cells induces basal-like features

**Fig. 4:**
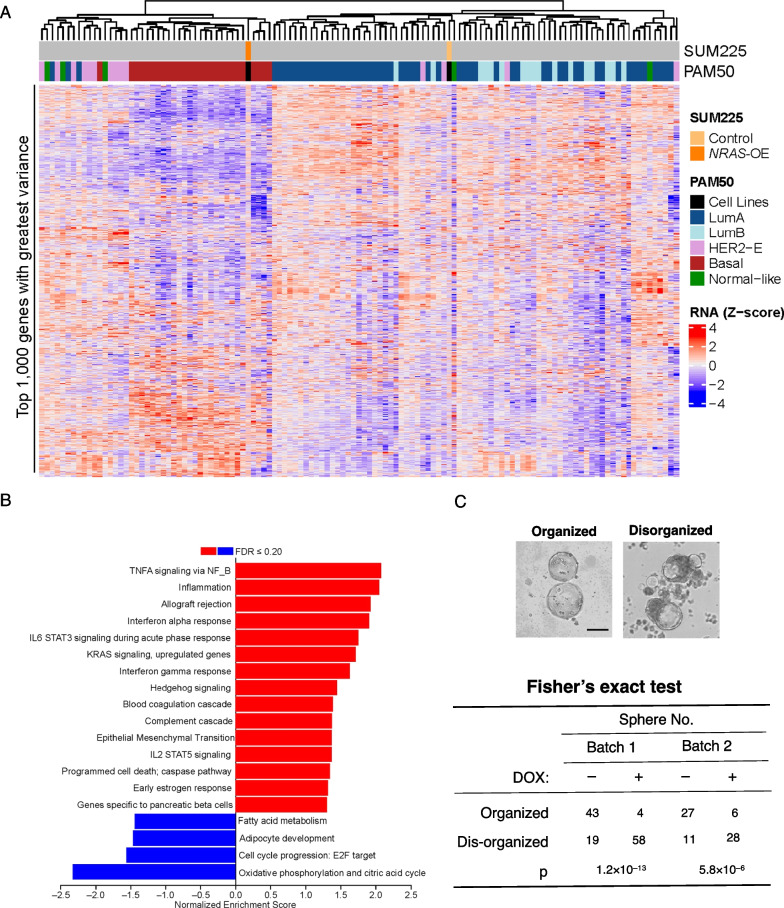
*NRAS* overexpression induces basal-like features in a human luminal DCIS model SUM225. **A** SUM225 cells overexpressing N-Ras and the counterparts carrying the vector control were cultured for 5 weeks. The RNA-seq data generated from these SUM225 cells and from the same CPTAC breast cancer cohort as in Fig. 3B were combined, and then, the top 1000 most variable genes across combined samples were used to perform unsupervised hierarchal clustering. **B** Gene Set Enrichment Analysis on Hallmark gene sets was performed using signed –log10 *p* values from limma results. Gene sets represented as red bars are up-regulated, while blue bars are down-regulated in *NRAS-*overexpressing SUM225 cells. **C** SUM225 cells carrying a DOX-inducible vector to overexpress *NRAS* were seeded with or without DOX in low attachment dishes for mammosphere formation. Normal spheres that are mostly round with a smooth boundary were counted as normal (top). The disorganized spheres usually have irregular shapes with cell masses that protrude from the boundary. Bar = 200 µm. Whether the portions of disorganized spheres are more common in NRAS overexpressing cells (+ DOX) in two separate experiments was examined by Fisher’s exact test (bottom)

We performed the converse experiment by stably overexpressing *NRAS* [15] in a luminal DCIS model cell line SUM225 (Fig. 1B). RNA-seq data (Additional file 4: Table S3) revealed that the gene expression patterns in the N-Ras overexpressing SUM225 cells were mostly clustered with those from the basal-like tumors in the same CPTAC patient cohort as described above (Fig. 4A). In contrast, SUM225 cells carrying the vector control displayed a mostly luminal-like gene expression pattern as expected.

To further analyze what biological activities were perturbed by NRAS expression, we performed GSEA (Additional file 4: Table S3, Fig. 4B). The data showed that the top hallmark pathways induced after NRAS overexpression are enriched with immune/inflammatory pathways, which is similar to what has been reported previously in basal-like breast tumors [15]. These results agree with the concept that NRAS overexpression can promote a shift from luminal to basal-like properties. Furthermore, an epithelial to mesenchymal transition (EMT) signature was among the pathways significantly correlated with NRAS expression (Fig. 4B). This is a feature that is usually observed in cells with more invasive potentials, but future validation is needed.

We also seeded *NRAS*-overexpressing SUM225 cells for mammosphere formation, in which the number and size of the sphere are typically assessed for stem-like properties. We did not observe any major difference in the number of spheres with respect to NRAS expression status. In the absence of DOX, SUM225 cells mostly form organized mammospheres that are round (Fig. 4C). These mammospheres have low cell density in the middle, which is surrounded by a distinct membrane boundary. In contrast, the *NRAS-*overexpressing SUM225 cells (+ DOX) formed disorganized structures in which dense cell masses appear to spread out easily. These subtype-specific mammosphere features agree with those observed using human breast cancer cells and mouse mammary cells [34, 35]. The size of these abnormal spheres is usually larger than the round one regardless of NRAS expression status.

## Discussion

The molecular mechanisms that drive a transition from DCIS to IDC are currently largely unknown, leading to overtreatment. Our analyses of patient samples illustrate that *NRAS* expression levels in DCIS correlate with invasiveness, as well as with biomarkers associated with high risks for progression such as low ER and high Ki67 [28, 36, 37]. Moreover, *NRAS* mRNA levels and NRAS gene expression signature correlate with basal-like properties in DCIS. *NRAS* silencing in a basal-like DCIS cells can induce luminal gene expression patterns; conversely, *NRAS* overexpression promotes basal-like gene signatures in luminal DCIS cells. These results support our conclusion that *NRAS* may be overexpressed by clonal subpopulations within DCIS that drive the emergence of basal invasive breast cancers.

Bergholtz and colleagues [38] analyzed 57 pure DCIS and 313 invasive breast cancer by gene expression, DNA methylation, and DNA copy number. Their studies found that the most significant differences were observed between basal-like DCIS and basal-like IDC. Basal-like DCIS showed lower correlation with core basal-like gene signature, as compared to basal-like IDC. Interestingly, basal-like DCIS showed higher correlation with luminal-A subtype, higher degree of differentiation, and lower proliferation rate. Furthermore, basal DCIS showed fewer copy number aberrations compared to basal-like IDC. These data suggest that basal-like IDC may evolve from non-basal-like DCIS, such as a subset of luminal DCIS in which *NRAS* is overexpressed.

We have shown that N-Ras itself can be targeted for degradation as a potential therapeutic strategy [39]. Furthermore, N-Ras appears to act via JAK2 to turn on IL8 expression in basal-like breast cancer [15]. Therefore, another potential therapeutic strategy may be to block Jak2 activation and or to use an IL8 blocker at the stage of DCIS. Reparixin is an orally bioavailable inhibitor for IL8 receptors, CXCR1/CXCR2. Reparixin was recently evaluated in a Phase II clinical trials in combination with Paclitaxel for patients with metastatic TNBC [40]. While the primary endpoint of prolonged progression-free survival was not met, the expression of *NRAS* may be a potential biomarker of response in these patients in future studies.

## Conclusion

After a DCIS diagnosis, a key problem is whether to treat the patients given the fact that only up to 40% of untreated DCIS cases will progress to invasive disease. Our study is the first to demonstrate NRAS as a potential driver of DCIS transition to invasion, and both NRAS and its downstream effector  are druggable. Despite our promising results with a small sample size, future studies evaluating a larger patient cohort and efficacy studies using NRAS targeting agents to inhibit progression to IDC should be pursued.

## Supplementary Information

**Additional file 1: Table S1.** Pathological, Biomarker and Demographic Characteristics of the patients in the TMAs.

**Additional file 2.** Figure S1, related to Figure 3 — measuring NRAS and KRT8 expression over time after gene silencing.

**Additional file 3**: **Table S2**.  NRAS-silencing induced a switch from basal to luminal gene expression pattern in SUM102PT cells as measured by RNA-seq.

**Additional file 4**: **Table S3**. NRAS overexpression induces basal-like features in a human luminal DCIS model SUM225 cells.

## Data Availability

RNA-seq generated by this study for SUM102PT and SUM225 cell lines has been deposited in the GEO public database, and access will be given when the paper is ready for publication. Processed Log2 RSEM upper quantile normalized data needed for analysis are already in Additional file 3: Table S2 and Additional file 4: Table S3.

## Abbreviations

CPTAC: Clinical proteomic tumor analysis consortium
DCIS: Ductal carcinoma in situ
DOX: Doxycycline
FFPE: Formalin-fixed paraffin embedded
FISH: Fluorescence in situ hybridization
GSEA: Gene Set Enrichment Analysis
IBC: Invasive breast cancer
IDC: Invasive ductal carcinoma
KD: Knock down
OE: Overexpression
TMA: Tumor microarray
TNBCs: Triple negative breast cancers
