# Supplementary material for: Elevated NRAS expression during DCIS is a potential driver for progression to basal-like properties and local invasiveness
Source: Breast Cancer Res. 2022 Oct 18;24:68. doi: 10.1186/s13058-022-01565-5 (PMC9578182; doi:10.1186/s13058-022-01565-5)
Supplement: Supplementary file 2 — Additional file 2. Figure S1, related to Figure 3 — measuring NRAS and KRT8 expression over time after gene silencing. [file 13058_2022_1565_MOESM2_ESM.docx]

**Supplementary Information**

**Elevated *NRAS* expression during DCIS is a potential driver for progression to basal-like properties and local invasiveness**

Ze-Yi Zheng^1, 2*^, Hanan Elsarraj^3*^, Jonathan T. Lei^1^, Yan Hong^3^, Meenakshi Anurag^1^, Long Feng^1, 4^, Hilda Kennedy^1^, Yichao Shen^1^, Flora Lo^1^, Zifan Zhao^1, 5^, Bing Zhang^1^, Xiang H. -F. Zhang^1, 6^, Ossama W. Tawfik^7^, Fariba Behbod^3¶§^, Eric C. Chang^1, 6¶§^

^1^Lester and Sue Smith Breast Center and Dan L. Duncan Comprehensive Cancer Center, Baylor College of Medicine, Houston, TX 77030, USA.

^2^Department of Medicine, Baylor College of Medicine, Houston, TX 77030, USA.

^3^Department of Pathology and Laboratory Medicine, University of Kansas Medical Center, Kansas City, KS 66160, USA.

^4^Department of Pathogenic Organism Biology, Henan University of Chinese Medicine, Zhengzhou, P.R. China.

^5^Cancer Cell Biology Graduate Program, Baylor College of Medicine, Houston, TX 77030, USA.

^6^Department of Molecular and Cellular Biology, Baylor College of Medicine, Houston, TX 77030, USA.

## ^7^MAWD Pathology Group**,** St. Luke’s Hospital, Lenexa, KS 66215, USA

*These authors contributed equally; ^¶^These authors contributed equally.

^§^Correspondence: [echang1@bcm.edu](mailto:echang1@bcm.edu), [fbehbod@kumc.edu](mailto:fbehbod@kumc.edu).

**Figure S1, related to Figure 3.** SUM102PT cells carrying a DOX-inducible shRNA against *NRAS* were seeded with or without DOX and cultured up to 4 months. Cells were harvested at the indicated time points, and *NRAS* and *KRT8* mRNA levels were measured by qPCR. mRNA levels were normalized to those of the −DOX control cells. d, days; w, weeks; m, months.
